# A scoping review of life skills development and transfer in emerging adults

**DOI:** 10.3389/fpsyg.2023.1275094

**Published:** 2023-11-16

**Authors:** René Tanious, Pierre Gérain, Wolfgang Jacquet, Elke Van Hoof

**Affiliations:** Faculty of Psychology and Educational Sciences, Vrije Universiteit Brussel, Brussels, Belgium

**Keywords:** life skills, life skills development, life skills transfer, emerging adults, scoping review

## Abstract

Emerging adulthood is a unique and sensitive life period during which most psychiatric conditions emerge. Development of life skills and transfer of these skills between contexts may be considered essential for transitioning from emerging adulthood to young adulthood. In the present scoping review of 83 articles published between 2010 and 2022, we address the state of research on life skills development and transfer in emerging adults aged 18–30. Specifically, we were interested in how life skills are defined, which specific life skills are proposed and how life skills transfer is conceptualized for this age group, with an emphasis on cognitive, personal, and interpersonal components. The results show that only a small minority of the reviewed studies defined the term life skills and an even smaller number mentioned the concept of life skills transfer, with only one study actually mentioning transfer components. Life skills research for emerging adults strongly focuses on vulnerable populations (e.g., developmental and contextual vulnerabilities, and sexually transmittable infections treatment and prevention) with the five most frequently mentioned life skills being budgeting/ finances, communication, problem-solving, decision-making, and emotional regulation. We discuss these results in light of future research directions and the lessons learned for life skills development and transfer research in emerging adults.

**Systematic review registration:**

All analyzed articles are available at: https://osf.io/gmk8w/.

## Introduction

Emerging adulthood is a unique and sensitive life period during which most psychiatric conditions emerge. The median age of onset for many major mental health conditions such as anxiety disorder, personality disorders, substance abuse disorders, and depressive disorders ranges from the late teens to the late twenties (Kessler et al., 2007; Solmi et al., 2022). The World Health Organization (WHO) estimates that during the COVID-19 pandemic, vital services in the mental health sector were either severely disrupted or temporarily halted in 93% of the countries worldwide, while demand for them was just increasing (UNICEF, 2021). A knowledge mapping analysis of 8,856 articles published worldwide during the pandemic revealed that the most common psychological problems were anxiety and depression (Zhou et al., 2022). Research into the mental health effects of the pandemic further shows that adolescents who were approaching the median age range of onset for major mental health disorders, as well as young adults already in this age group were particularly affected by the pandemic (Salmon et al., 2022). More specifically, it has been found that persons aged 20 to 29 years were especially susceptible to increased feelings of loneliness and associated depressive symptoms (Lee et al., 2020) as well as COVID-19 related post-traumatic stress disorder (Benatov et al., 2022). These conditions do not simply fade away post-pandemic and they may become chronic without adequate support. Thus, it is important to develop programs post-pandemic to address the generally worsened mental health in this age group. Life skills have been suggested to promote mental health in this age group in general (Sobhi-Gharamaleki and Rajabi, 2010; Savoji and Ganji, 2013) and in the context of COVID-19 in particular (Velasco et al., 2021). A general framework for life skills education and its importance has been developed by WHO and UNICEF.

### Who and UNICEF life skills framework

In 1998, a United Nations inter-agency meeting between WHO and the United Nations Children’s Fund (UNICEF) was held with the aim “to generate consensus among United Nations agencies as to the broad definition and objectives of life skills education and strategies for its implementation” (WHO, 1999, preamble). Following the meeting, WHO (1999) published a document detailing the main conclusions. While admitting that the term is “open to wide interpretation” (p. 3), WHO identified five key areas of life skills that are sought to be relevant across cultures: decision-making and problem-solving, creative and critical thinking, communication and interpersonal skills, self-awareness and empathy, and coping with emotions and stress. These key areas were grouped under the term psychosocial skills by WHO. More recently, UNICEF (2012) admitted that life skills was still a highly elastic concept that lacks a full and widely accepted definition. To fill this void, UNICEF stated that much discourse points “toward their importance to our protection and well-being and our ability to live productive, meaningful and fulfilling lives” (p. 7). Building upon the work by WHO and other agencies, UNICEF then identified three broad categories of generic life skills: cognitive (critical thinking, problem-solving, decision-making), personal (awareness, drive, self-management), and interpersonal (communication, negotiation, cooperation, teamwork, empathy, and advocacy). These life skills “are considered generic and empowering in their own right” (p. 9).

### Existing life skills teaching research: a brief overview

Regarding the existing frameworks, much research has been conducted within the UNICEF and WHO on how life skills can be taught. UNICEF (2012) itself highlights the role of the parental home and family life, stating that this “environment has been identified as an important factor in establishing the foundations for skills, attitudes and values relating to society” (p. 11). At the same time, UNICEF acknowledged that wider societal structures such as prevailing religious and cultural attitudes play a role.

Such structures and attitudes may also manifest themselves in the classroom, which constitutes another context in which life skills teaching has been studied. For example, Wurdinger and Rudolph (2009) praise a student-centered approach consisting of project-based learning where high school students can determine their own projects and work at their own pace. According to the authors, such an approach allows for developing life skills such as (time) management, problem solving, and responsibility. In their case study, Wurdinger and Rudolph found that 50% of the alumni from a school implementing such an approach graduated from college, which was considerably higher than the US national average. Other activities that have been suggested for teaching life skills in the classroom include classroom discussions, debates, brainstorming, educational games, and story-telling (Prajapati et al., 2017). Within the school environment, it has also been researched how life skills can be developed through school-based sport programs. For example, Danish et al. (2005) developed a sports-based life skills program in schools based on physical and mental skills improvement, emphasizing that “sport-based life skills programs implemented in schools are designed to help the student learn both sport and life skills. Therefore, what is learned in the sport must be able to be transferred to nonsport settings” (p. 54).

Research about the teaching and transfer of life skills through sports has received much attention in general. Based on a plethora of prior research on life skills development in sports (e.g., Turnnidge et al., 2014; Bean et al., 2018; Camiré et al., 2022; DeJaeghere and Murphy-Graham, 2022), Camiré (2022) proposed a two continua model of how sports coaches can teach life skills. The model distinguishes between implicit and explicit approaches along one axis, and normative and transformative approaches along the other axis. According to Camiré’s model, sports coaches can teach life skills along a continuum from implicit to explicit. Implicit coaches do not intentionally teach life skills, but may still do so under the premise that the sport context with clear rules provides a healthy learning environment. Explicit coaches intentionally focus on the teaching of life skills by providing initiatives to practice and discuss life skills. Along the other axis, normative life skills teaching prioritizes compliance to dominant sociocultural ideals whereas coaches using an transformative approach focus on life skills for social change to enable youth to live well outside the sport context. Combining the two axes, transformative explicit approaches are most benevolent according to Camiré.

### The concept of life skills transfer

Once life skills have been acquired, an important feature is the application of said skills in different settings. Complementing the previously mentioned definition of life skills by UNICEF (2012), other sources highlight this transfer aspect of life skills. For example, Morowatisharifabad et al. (2019) define life skills as “strategies that enable people to live a successful life *in different environments*” (p. 9623, emphasis added). Similarly, Camiré (2022) asserts that “life skills refer to a person’s ability to do life well through the successful completion of everyday tasks and a meaningful engagement *at school, at work, at home and in the community*” (p. 1, emphasis added). Within the theoretical framework of the present study, we define life skills transfer as the application of life skills in settings other than the one(s) where they were originally acquired. For example, many of the skills developed in a sports setting (e.g., problem-solving, communication) are transferable to other life domains (Papacharisis et al., 2006).

In addition to learning and developing life skills, this transfer process of skills itself is another topic of scholastic inquiry: “A parallel line of research, called transformative learning, finds that transfer is a multi-dimensional experience that includes cognitive processes” (Jacobs and Wright, 2021, p. 380). Drawing from the context of sports, Jacobs and Wright further outlined that the evidence of transfer to other life domains is limited. Similarly, Holt et al. (2017) conducted a meta-analysis of positive youth development through sports studies and concluded that “there was relatively little information about explicit pedagogical strategies designed to promote transfer. In fact, there were several examples that showed that transfer could occur implicitly, without deliberate attempts” (p. 34). As this highlights, how exactly the transfer process takes place, for example in terms of cognitive, personal, and interpersonal components as outlined in the UNICEF (2012) framework, appears not to be well researched.

### Emerging adulthood as a distinct developmental stage: the rationale for the current study

Both the WHO and UNICEF framework, as well as much of the literature presented in the previous paragraphs, focus on life skills development and/or transfer among children and adolescents, probably under the assumption that “life skills are essential for the successful transition from dependent childhood to independent adulthood” (Donnellan and Mathews, 2021, p. 547). However, the timing and shape of transition into adulthood has changed drastically since the second half of the 20th century. Specific markers of transitioning to adulthood such as leaving the parental home, entering full-time employment, marrying, and becoming parents occur much later in life: “Any of these transitions occurred at or near the time of reaching legal adulthood in the 1960s and 1970s, they often occur in people’s mid- and late-20s—or later—today (Ribar and Wong, 2022, p. 157). In describing this trend, Arnett (2000, 2007) proposed that the prolonged transition into adulthood presents a distinct developmental stage, which he coined emerging adulthood, beginning after adolescence and ending with reaching young adulthood. In industrialized nations, emerging adulthood appears at a later age and over a longer period (Arnett, 2000). This does of course not invalidate the notion that “given the role of life skills as a strong catalyst for the development of positive behavior, building life skills in the early years of life will help children navigate their social and emotional challenges” (Nasheeda et al., 2019, p. 375). Life skills development and transfer remains important for children and adolescents.

At the same time, given the distinct nature and challenges of emerging adulthood, it is of particular interest to investigate the current state of research regarding life skills development and transfer in this particular age group. Young persons in emerging adulthood are legal adults, yet not fully autonomous and often dependent on their families (Ribar and Wong, 2022). As further outlined by Arnett et al. (2014), emerging adulthood is a sensitive life period associated with instability, feeling in-between, and identity explorations. Adequate support systems for emerging adults may be lacking: “It could be argued that organizing mental health services into separate child and adult systems does not serve the transition-aged population well” (Davis and Koroloff, 2006, p. 55). The empowering nature of life skills, as highlighted by the UNICEF framework, may make them suitable leverage to better manage the transition to young adulthood. Accordingly, it has been found that life skills can act as important enablers for mental health help-seeking behaviors as well as developing adequate coping strategies in adolescents and emerging adults (Hellström and Beckman, 2021). Thus, it may be argued that the development and transfer of life skills -whether it concerns life skills learned at this age or earlier in life- for this age group may be of particular importance for positive mental health development in emerging adults.

### Research questions

To gain a better understanding of the concept of life skills for emerging adults following from the theoretical frameworks, we aim to answer the following research questions:

1. How are life skills defined in the literature for emerging adults?
2. Which life skills are proposed in the literature for emerging adults?
3. What is the state of research regarding life skills transfer in emerging adults?
4. What are the cognitive, personal, and interpersonal aspects of life skills transfer among emerging adults discussed in the literature?

## Method

Following the guidance by Munn et al. (2018) on choosing an appropriate review method, a scoping review approach was chosen for three reasons. First, scoping reviews can be used to clarify key concepts and definitions in the literature which is important to assess whether consensus on a life skills framework has been reached. Second, scoping reviews are best suited to identify and map the available evidence in a given research domain and, by extension, to identify knowledge gaps which can catalyze future research. Third, scoping reviews can be used to identify the types of available evidence in a given field (e.g., intervention versus non-intervention studies), which is important for assessing the level of evidence of life skills studies.

### Justification for publication period selection

A systematic search of the life skills literature from 01/01/2010 to 10/31/2022 was conducted. The reasons for selecting this time period were threefold. The first reason for the selection of this time period was the publication of the UNICEF framework on life skills in 2012, as outlined in the introduction. The publication of that framework may well have had an impact on subsequent research into life skills.

Second, a preliminary search of the PubMed library using the search term “life skill*” was conducted. With few exceptions, the number of publications has been rising steadily from 2010 onwards. In fact, the number of publications on the subject has more than trebled between 2010 and 2022 (see Supplementary Appendix A).

Third, this increase in the number of publications may be attributed to technological advancements happening around that period, most notably internet becoming widely adopted. In 2010, the number of internet users surpassed two billion (International Telecommunications Union, 2010). By 2014, around 40% of the world population was using the internet, with a sharp divide between developing and developed countries with 32 and 78%, respectively (International Telecommunications Union, 2014). The transformation of an analog society to a digital one expands the concepts of life skills to the digital arena as well. In their systematic review about the outcomes of gaining digital skills, Livingstone et al. (2021) found that digital skills are most frequently discussed in relation to life skills. Similarly, van Laar et al. (2017) presented a systematic review in which life skills, digital skills, and learning skills are distinct but intrinsically linked skills.

### Search query and data bases

The search query was refined through several iterations within each database using different combinations of keywords and Boolean operators. Synonyms for the included terms were uncovered by scanning abstracts of articles appearing in earlier iterations as well as by checking dictionaries. The generic search query used was (“life skill*” OR “lifeskill*”) AND (“young adult*” OR “emerging adult*” OR “young people”). This query was initially developed for Web of Science and subsequently modified to meet the specific requirements of each data base. In addition to Web of Science, Scopus, PsycInfo, and PubMed were searched. According to Gusenbauer and Haddaway’s (2020) extensive study assessing the eligibility of different academic databases, these four are suited for use as primary search engines in systematic reviews. These search engines meet important quality markers, most notably functionality of Boolean operators and reproducibility across time and locations. The exact search query per database can be found in the Supplementary Appendix B. The search query was set to search title and abstract, as well as keywords (the last only applicable to Scopus).

### Inclusion and exclusion criteria

**Figure 1:**
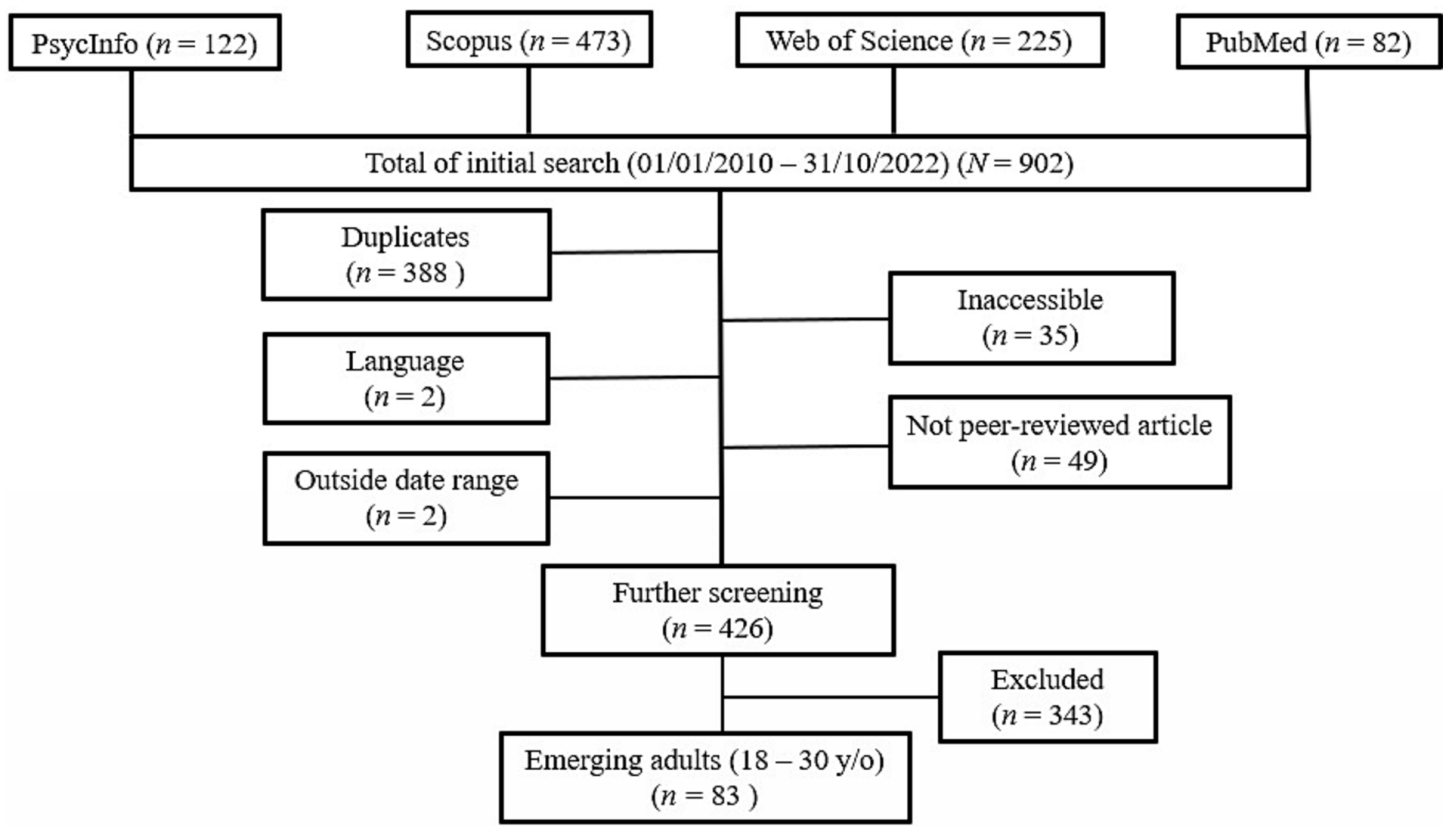
Study inclusion flowchart.

All articles had to meet the following criteria in order to be eligible for inclusion in the present scoping review. Articles had to be published between 01/01/2010 and 10/31/2022. To determine this, the date of first online publication was used. In addition, all articles had to be written in English. The age range was set to 18 to 30 years old because research indicates that by age 30 the rapid changes of emerging adulthood have typically stabilized in the general population (Davis and Koroloff, 2006). Articles falling in- and outside of this age range (e.g., 16–25) were decided on a case-by-case basis on the criterion that they either mention the transition to adulthood or -if applicable- the mean age falling within the specified age range. The study inclusion flowchart can be found in Figure 1.

In a first step, all duplicates between databases were removed, leaving 514 unique references. Subsequently, articles were checked for the following exclusion criteria: language other than English, inaccessibility, outside date range, and not peer-reviewed. This left 426 articles in the initial sample for further screening, of which 83 fell within the age range of emerging adulthood and were included in the final sample. All articles included in the final sample can be found in the Open Science Framework at https://osf.io/gmk8w/.

### Interrater reliability for inclusion

Each article in the initial sample was assigned a unique identification number. 10% of all articles (*N* = 43) were selected by randomly drawing from the pool of identification numbers in R using the *choose* function. In case of difference of opinion, the articles were discussed between the first and second author until agreement had been reached on the in- or exclusion of each article. The remaining articles were then coded by the first author.

## Results

Table 1 presents general characteristics of the reviewed studies. Overall, only two-thirds of all studies (66.27%) mentioned specific life skills and less than one in five studies (18.07%) actually defined the term life skills. The studies were almost evenly split between mentioning (49.60%) and not mentioning (50.40%) the transition to adulthood explicitly. Transfer of life skills was mentioned infrequently in only 9.64% of the reviewed studies and only one study mentioned specific transfer components.

**Table 1:** Key characteristics of included studies.

**Characteristic**	**Yes (*N*, %)**	**No (*N*, %)**
Intervention study	30 (36.14)	53 (63.86)
Defines life skills	15 (18.07)	68 (81.93)
Mentions specific life skills	55 (66.27)	28 (33.73)
Mentions transition to adulthood	41 (49.40)	42 (50.60)
Mentions transfer of skills	8 (9.64)	75 (90.36)
Mentions transfer components	1 (1.20)	82 (98.80)

Transfer of skills refers to the application of life skills in settings others than the one where they were originally acquired. Transfer components refers to cognitive, personal, and interpersonal aspects necessary for life skills transfer.

### Life skills definitions

Within the studies that defined life skills, a distinction can be made between studies following an existing framework and studies presenting their own definition. Of the 15 studies defining life skills, nine (60.00%) used the frameworks of UNICEF and WHO presented in the introduction (Okun et al., 2016; Jordaan et al., 2018; Ingram and Dorsett, 2019; Kingsnorth et al., 2019; Duff et al., 2020; Atif et al., 2021; Hellström and Beckman, 2021; Pearson et al., 2021; Pletschko et al., 2023).^1^ These studies covered a diverse range of topics, such as for example preparing for parenthood in low and middle-income countries (Atif et al., 2021), acquiring life skills with congenital total blindness (Ingram and Dorsett, 2019), and psychosocial support programs for young adult childhood cancer survivors (Pletschko et al., 2023).

Four studies (26.67%) used their own definitions of life skills. Those definitions cannot easily be categorized, but two definitions highlight relationships with others. One definition highlights the individual in the community: “life skills were those deemed necessary for an individual to exist in an inclusive community, including living, working, and having fun” (Bouck et al., 2016, pp. 342–343), whereas another highlights individual needs and relationship satisfactions: “Life skills refer to youths’ ability to provide for their needs, to feel comfortable with themselves and be satisfied with relationships with significant others in the family, community and society (Maluccio et al., 1990)” (Sulimani-Aidan and Melkman, 2022, p. 1484). Another definition highlights the individual and life skills transfer: “Life skills in this context are defined as cognitive, emotional and behavioral skills that can be learned in one context (e.g., through sport) and transferred to and used effectively in other contexts (such as education […])” (Brooke et al., 2019, p. 294). Finally, Mmusi and van Breda (2017) define social skills in addition to independent living skills as “specific behaviors that an individual exhibits to perform competently on a social task (e.g., active listening skills, reciprocal communication, ignoring, etc.)” (Holosko, 2015; as cited Mmusi and van Breda, 2017, p. 351).

Two studies (13.33%) drew from existing frameworks other than WHO or UNICEF. Güçlü et al. (2022), investigating emerging adults’ perceived life skills, used three existing frameworks: Assessment and Teaching of 21st Century Skills [ATCS] (Binkley et al., 2011), Organization for Economic Co-operation and Development [OECD] (Ananiadou and Claro, 2009), and Partnership for 21st Century [P21] (2009). The ATCS framework highlights ways of thinking, ways of working, work tools, and life on earth. OECD mentions effective use of tools, interaction in heterogenous groups, and autonomy. Finally, P21 uses three overarching topics of life skills: learning and innovation skills, information, media and technology skills, and life and career skills. The other study in this category drew from a framework pre-dating the WHO and UNICEF frameworks. Currie et al. (2012) conducted an exploratory study examining the relationship between life skills and academic success. In doing so, the authors drew on Brooks (1984) taxonomy of life skills which delineates four areas of life skills: interpersonal communication/human relations, problem-solving/decision-making, physical fitness/health maintenance, and identity development/purpose in life.

### Topics and specific life skills mentioned

Of the 12 topics that emerged, the three most frequent ones accounted for nearly two-thirds (66.27%, *N* = 55) of the reviewed studies: developmental vulnerabilities (mental health issues/physical, developmental, and intellectual disabilities) (*N* = 24), sexual behavior (including prevention and treatment of sexually transmittable infections [STIs] and LGBTQ+ needs) (*N* = 17), and contextual vulnerabilities (foster care/homelessness) (*N* = 14). The other topics that emerged were: university students (*N* = 5), substance abuse reduction and prevention (*N* = 4), developing world/poverty (*N* = 3), survivor of life threatening disease (*N* = 3), young offenders/prison experience (*N* = 3), sports (*N* = 2), ethnic minorities (*N* = 2), employability (*N* = 2), and miscellaneous (*N* = 4). In relation to the number of articles, the sexual behavior category mentioned relatively few life skills, whereas the opposite holds true for the category developing world/poverty. Within the most frequent category of developmental vulnerabilities, a broad array of life skills was proposed ranging from communication and budgeting to managing contraception and self-actualization. Across all studies, the five most mentioned life skills were budgeting/finances (*N* = 19), communication (*N* = 18), problem-solving (*N* = 13), decision-making (*N* = 12), and emotional regulation (*N* = 11). However, these results are strongly context-specific. Therefore, Table 2 offers an overview of the proposed life skills per topic and Table 3.

**Table 2:** Topics and proposed life skills of reviewed studies.

**Topic**	***N* (%)**	Proposed life skills (N*)
Developmental vulnerabilities (mental health issues/physical, developmental, and intellectual disabilities)	24 (28.92)	Communication (6), budgeting/finances (5), emotional regulation (5), problem-solving (5), social skills (5), managing transportation/public transport (4), decision-making (3), motivation (3) self-advocacy (3), (self-) confidence (3), time management/calendaring (3), coping skills (2), interpersonal relationship skills (2), goal-setting (2), independence (2), adaptive behavior, assertiveness, awareness of accessibility policies, choosing age-appropriate clothing, cooking, creative and critical thinking, daily living skills, disclosure, empathy, executive functioning skills, externalizing, future planning, housing, internalizing, intrapersonal development, managing contraception, mobility, personal hygiene, resilience, self-actualization, self-care, self-determination, self-awareness, self-efficacy, self-expectations, self-management
Sexual behavior (including prevention and treatment of STIs and LGBTQ+ needs)	17 (20.48)	Communication (3), self-esteem (3), coping, knowledge of safe sexual practices, self-efficacy, self-concept, stress management, social support management, transgender-related stress and stigma awareness
contextual vulnerabilities (Foster care/homelessness)	14 (16.87)	Budgeting/finances (13), cooking/food preparation (6), find/manage housing (6), independence (4), planning education/study (4), social/family relationship management (4), decision-making (3), job search and maintenance (3), set and pursue goals (3), career planning (2), communication (2), accepting consequences and criticism, daily living, driving, following instructions, honesty, interview skills, looking forward, manage emotions, medication management, mental and physical health management, paying taxes, peer pressure resistance, permanency, personal care, refraining from illegal substances, resume writing, safe sex awareness, self-care, self-control, self-advocacy, taking responsibility, understanding trauma
University students (diverse topics)	5 (6.02)	Decision-making (2), problem-solving (2), autonomy, balancing work and well-being, critical thinking, creativity, emotional regulation, financial planning, health maintenance, human relationships, identity development/purpose in life, real estate planning
Substance abuse reduction and prevention	4 (4.82)	Assertiveness (3), decision-making (2), stress management (2), anger management, communication, coping skills, goal-setting, problem-solving, refusal skills
Developing world, poverty	3 (3.61)	Communication (2), coping with emotions (2), coping with stress (2), critical thinking (2), problem-solving (2), awareness, assertiveness, creativity, decision-making, emotional awareness, empathy, goal-setting, interpersonal skills, self-regulation
Survivor of life threatening disease	3 (3.61)	Communication (2), independence (2), collaborative skills, community living skills, conflict management, motor skills, personal living skills, self-management, self-perception, social interaction
Young offenders, prison experience	3 (3.61)	Anger management, communication, coping, decision-making, emotion regulation, problem-solving, vigilance, self-awareness, stress management
Employability	2 (2.41)	Applied mathematics, cognitive skills, communication, critical thinking, future vision, information reading, interpersonal relationships, problem-solving, self-efficacy, self-management self-reflection, social skills
Sports	2 (2.41)	Cooperation, mental toughness, self-discipline, self-realization
Ethnic minorities	2 (2.41)	Assertiveness, budgeting, cooking, child caretaking, creativity, household, openness, patience, persistence, pro-activity, self-confidence
Miscellaneous	4 (4.82)	Self-esteem (2), leadership ability (2), autonomy, communication, conflict resolution, coping, comfortable with self, empathy, financial maintenance, health maintenance, household maintenance, independent living, interpersonal manners, intimacy, knowledge summarization, maintaining a job, needs provision, planning, positive thinking, problem-solving, psychosocial well-being, relationships satisfaction, resilience, risk behavior avoidance, self-competence, work skills

*Only indicated if *N* > 1.

**Table 3:** Study characteristics of the most common topics.

**Topic**	**Characteristic**	**Yes (*N*, %)**	**No (*N*, %)**
Developmental vulnerabilities	Intervention study Defines life skills Mentions specific life skills Mentions transition to adulthood Mentions transfer of skills Mentions transfer concepts	6 (25.00) 6 (25.00) 18 (75.00) 13 (54.17) 6 (25.00) 0	18 (75.00) 18 (75.00) 6 (25.00) 11 (45.83) 18 (75.00) 24 (100)
Sexual behavior	Intervention study Defines life skills Mentions specific life skills Mentions transition to adulthood Mentions transfer of skills Mentions transfer concepts	10 (58.82) 0 3 (17.64) 4 (23.53) 0 0	7 (41.18) 17 (100) 14 (82.35) 13 (76.47) 17 (100) 17 (100)
Contextual vulnerabilities	Intervention study Defines life skills Mentions specific life skills Mentions transition to adulthood Mentions transfer of skills Mentions transfer concepts	3 (21.43) 1 (7.14) 13 (92.86) 14 (100) 1 (7.14) 1 (7.14)	11 (78.57) 13 (92.86) 1 (7.14) 0 13 (92.86) 13 (92.86)

For some topics, the proposed life skills appear to be more context-specific, whereas for other categories they seem to be more generic. For example, in the contextual vulnerabilities category the most frequently mentioned life skills were budgeting/finances (*N* = 13), cooking/food preparation (*N* = 6), and finding/managing housing (*N* = 6). These skills are particularly useful in the context of exiting foster care or transitioning from homelessness. Similarly, Assertiveness (*N* = 3), decision-making (*N* = 2), and stress management (*N* = 2) were the most frequently mentioned life skills in the substance abuse reduction and prevention category because these skills are needed to refrain from substance use, e.g., by being assertive toward one’s peers. On the contrary, out of the few life skills proposed in the sexual behavior category, the most frequently mentioned ones were communication (*N* = 3) and self-esteem (*N* = 3) which are rather generic, with communication also frequently mentioned in other topics.

### Study characteristics

Apart from the specific life skills mentioned, differences also exist between the various topics regarding the study characteristics (see Table 3). What stands out is that in the sexual behavior category only three studies (17.64%) mentioned specific life skills, none of the studies actually defined the term life skills, and none of the studies mentioned life skills transfer. At the same time, the sexual behavior category showed the highest percentage of intervention studies by a considerable margin compared to the other topics (58.82%). The term life skills was only somewhat frequently defined in the developmental vulnerabilities category (25.00%). The transition to adulthood was mentioned least frequently in the sexual behavior category (23.53%), whereas it was mentioned in all studies in the contextual vulnerabilities category and slightly over half of the studies (54.17%) in the developmental vulnerabilities category.

### Cognitive, personal, and interpersonal life skills taxonomy

**Figure 2:**
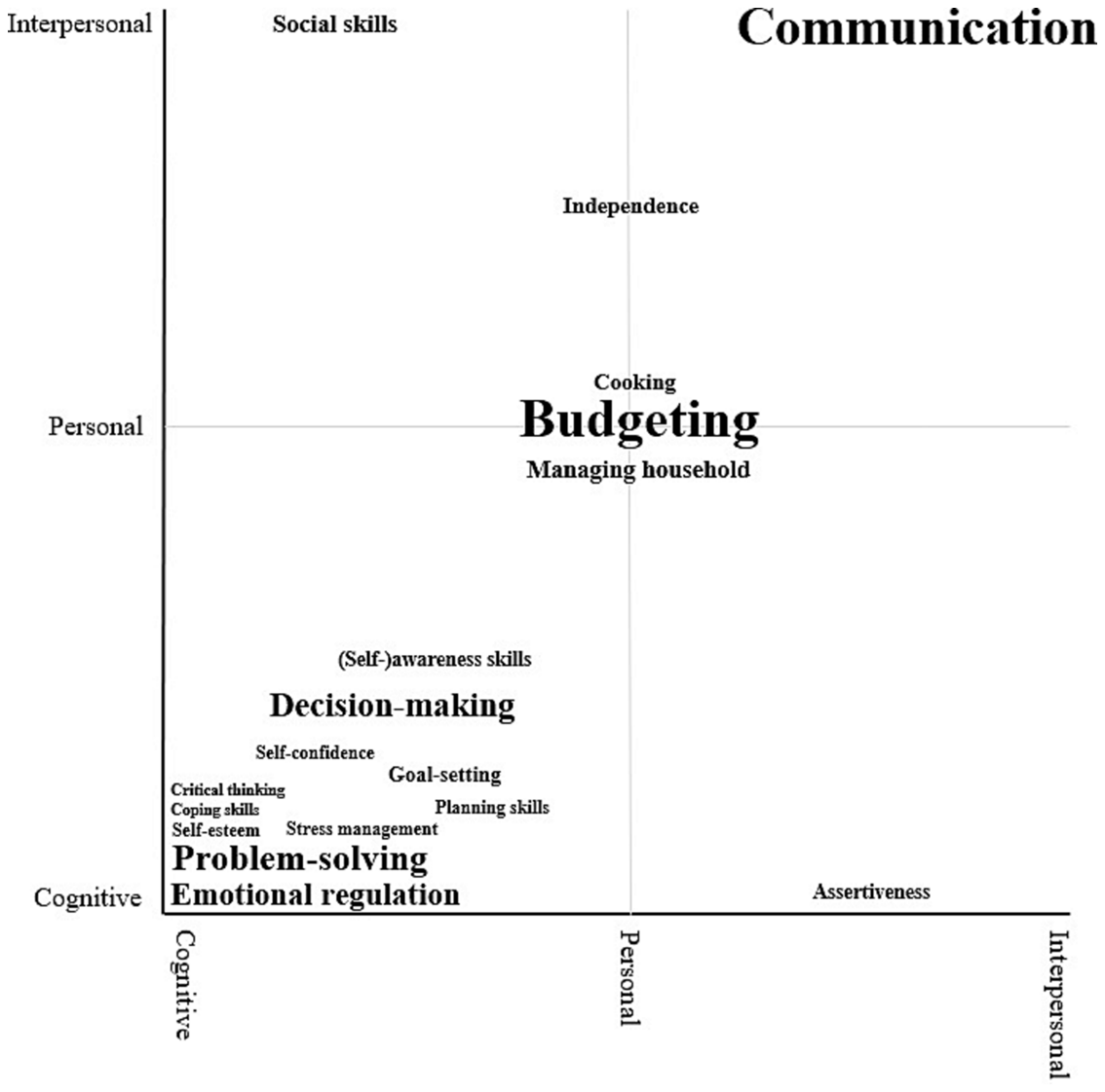
Taxonomy of life skills found in the scoping review along the dimensions of the UNICEF framework note. The size of each skills is proportionate to its frequency in the literature. To avoid excessive cluttering, only skills that have been found at least five times are included.

Regarding the distinction between cognitive, personal, and interpersonal life skills, UNICEF (2012) states that “there is no definitive list or categorization of the skills involved and how they might relate to one another. By their very nature such skills are largely intangible and difficult to isolate from the complex web of interactions […]” (p. 12). Figure 2 attempts to address this void while taking into account this web of interactions. Figure 2 presents a taxonomy of the life skills along the axes of the UNICEF framework: cognitive, personal, and interpersonal. Given the intangible nature of the skills, Figure 2 should be understood as flexible rather than rigid. Each of the life skills is proportionate in size to its frequency in the literature. To avoid cluttering, only life skills that have been found at least five times are included.

The lower left quadrant representing cognitive, personal skills shows the highest number of skills. This diverse range of life skills includes problem-solving, emotional regulation, decision-making, and goal-setting. These skills have been placed in the cognitive personal quadrant because they require cognitive capacity, but they are also personal in nature as they do not necessarily involve interactions with others. Coping skills, critical thinking, self-esteem, and problem-solving have been placed in the lower left corner as we consider them as requiring the most cognitive capacity. Emotional regulation has been placed in the lower left quadrant as well because emphasizing its cognitive nature, but more toward the personal axis as emotional regulation requires personal skills such as proactive self-management.

In terms of the number of life skills, the center of the taxonomy representing personal skills follows in frequency. These personal life skills include budgeting, managing a household, and cooking. While these skills of course also require cognitive capacity, they are more about executing. For example, planning and problem-solving are more cognitive skills, which can be used to execute these more personal skills.

Only communication falls strictly within the upper right corner representing interpersonal skills because it involves other persons per definition. Other life skills containing an interpersonal component include social skills and assertiveness. Social skills, which were mostly not further specified in the articles, have been placed on the interpersonal axis as well, but with a joint emphasis on the cognitive components they require, which in turn can help in effectively communicating. Finally, assertiveness has been placed on the cognitive axis with a joint emphasis on the personal and interpersonal components. Assertiveness requires strong cognitive capacity, but one needs to be assertive either toward oneself or other members of society, making this a personal and interpersonal skill as well.

### Life skills transfer and its cognitive, personal, and interpersonal aspects

Eight studies mentioned life skills transfer (Bal et al., 2016; Mmusi and van Breda, 2017; Brooke et al., 2019, 2020, 2022; Kingsnorth et al., 2019; Duff et al., 2020; Pletschko et al., 2023). Table 4 gives an overview of the characteristics and goals of these studies.

**Table 4:** Topics and characteristics of studies mentioning life skills transfer.

**Study**	**Context/goal**	**Intervention/Intervention development**	**Characteristics (Yes/No)**
Bal et al. (2016)	Vocational rehabilitation intervention for young adults with physical disabilities	Group sessions and individual coaching by an occupational therapist, psychologist, and a job-coach.	Defines life skills (N) Mentions specific life skills (Y) Mentions transition to adulthood (N) Mentions transfer concepts (N)
Brooke et al. (2022)	Enhancing functional recovery for young people recovering from first episode psychosis via sport-based life skills training	Various sporting activities, which were designed to promote physical activity, maximize social connectivity, and teach life-skills	Defines life skills (N) Mentions specific life skills (Y) Mentions transition to adulthood (N) Mentions transfer concepts (N)
Brooke et al. (2019, 2020)	Development of a sport-based life skills program for young people with first episode psychosis	None	Defines life skills (Y) Mentions specific life skills (Y) Mentions transition to adulthood (N) Mentions transfer concepts (N)
Brooke et al. (2019)	Perceived barriers to and enablers of sport participation for young people with first episode psychosis	Development of a sport-based life skills intervention for young people recovering from first episode psychosis	Defines life skills (N) Mentions specific life skills (Y) Mentions transition to adulthood (N) Mentions transfer concepts (N)
Duff et al. (2020)	Emotional literacy development for youth with disabilities	None	Defines life skills (Y) Mentions specific life skills (Y) Mentions transition to adulthood (Y) Mentions transfer concepts (N)
Kingsnorth et al. (2019)	Residential immersive life skills programs for youth with disabilities	Intensive summer program housed in a college residence that provides realistic experiences of living away from home for small groups of youth between 17 and 21 years of age	Defines life skills (Y) Mentions specific life skills (Y) Mentions transition to adulthood (Y) Mentions transfer concepts (N)
Mmusi and van Breda (2017)	Transfer of social skills from care into independent living	None	Defines life skills (Y) Mentions specific life skills (Y) Mentions transition to adulthood (Y) Mentions transfer concepts (Y)
Pletschko et al. (2023)	Psychosocial support program for young adult childhood cancer survivors	Development and evaluation of a targeted aftercare program with the goal of facilitating three important life skills: (1) self-perception, (2) social interaction and conflict management, and (3) self-conscious communication of support needs	Defines life skills (Y) Mentions specific life skills (Y) Mentions transition to adulthood (Y) Mentions transfer concepts (N)

Of the 15 studies that defined life skills (see Table 1), five studies mentioned life skills transfer (see Table 4). All studies mentioning life skills transfer also mention specific life skills, which is much higher than the 66.27% in the general study population (Table 1). Five out of the eight studies (62.50%) shown in Table 4 define life skills, which is also considerably higher than the 18.07% in the general study population. Similarly, 62.50% of these studies are intervention studies, whereas this percentage is only 36.14% in the general study population. The percentage of life skills transfer studies mentioning the transition to adulthood (50.00%) is nearly identical to the one in the general study population.

Of the eight studies that mentioned life skills transfer, only one (12.50%) mentioned specific transfer components (Mmusi and van Breda, 2017). Mmusi and Van Breda (2017), through an interview study, investigated the transfer of social skills learned in care to independent living in South Africa. As they note “there is little research to show whether and how these skills are transferred from the child care setting to young adulthood” (p. 350). Mmusi and van Breda found the following cognitive transfer components: recall, contextual flexibility, perceived usefulness, thoughtfulness, willingness, internalization, outcome expectation, perceived ability to use skill, and reflective living. In addition, they found personal challenges as a personal skill transfer component and upbringing as an interpersonal skill transfer component. Mmusi and van Breda point out that differences in context “impact not only the extent to which care-leavers feel support and protected […], but also how they utilize the skills they learned” (p. 354) and conclude that “for skills to be meaningful and useful, there has to be an interrelationship between application of a skill and real-life context” (p. 355).

## Discussion

The present scoping review was carried out under the premise that life skills are “essential skills particularly during the transition to adulthood” (Duff et al., 2020), with the main goal of assessing the state of research regarding life skills development and transfer in emerging adults. In light of the sensitivity of this transitory life stage with many uncertainties and fast changes, research on the specific factors relating to life skills development and transfer in this age group appears to be rather scarce. Overall, it appears that life skills is an umbrella term that is not clearly defined within the scientific literature.

What stands out is that a large majority of life skills literature focuses on vulnerable populations. This is manifested in the three most frequent topics being developmental vulnerabilities, sexual behavior, and contextual vulnerabilities. In addition, the methodological and conceptual qualities of some studies raise concerns. The sexual behavior category in particular, which included many studies over HIV/AIDS prevention and treatment, embodies several problematic characteristics. None of the studies in that category defined life skills, only a small minority mentioned specific life skills, and none of the studies discussed transfer of skills between contexts. With this category showing the highest number of intervention studies, the questions may then be raised on which theoretical frameworks the interventions were based and which specific life skills were targeted. Accordingly, few actionable insights can be derived on life skills development and transfer in typically developing emerging adults. As outlined in the results section, the most frequently mentioned life skills were budgeting/finances, communication, problem-solving, decision-making, and emotional regulation. In light of the emphasis on vulnerable populations in most studies, an immediate question is if these skills should also receive more attention in studies with typically developing emerging adults. These skills appear rather generic and a tentative answer to that question is that they could be used as impetus for studies with typically developing emerging adults as well. At the same time, lesser mentioned skills such as assertiveness, self-esteem, or future planning seem valuable outside of the context of vulnerable populations as well. Which specific skills to target with future interventions may therefore be a more context specific question, taking into account factors such as geographic locations and cultural backgrounds. In addition, the term life skills may need a more refined and updated definition to better inform future studies.

### Defining the term life skills

Based on the limited evidence available, few conclusions can be drawn about a preferred life skills definition. No dominant conceptual framework for life skills development and transfer in emerging adults materialized from the present scoping review. The vast majority of studies did not define the term life skills. In the few studies that did define life skills, the frameworks of WHO and UNICEF accounted for over half of the studies. It should, however, also be noted that even though the UNICEF and WHO frameworks were not mentioned frequently, some of the most frequently mentioned life skills (e.g., communication and decision-making) stem from the UNICEF framework, posing the question whether the UNICEF framework is used implicitly more often than it is explicitly referred to. Thus, based on the limited evidence available, the UNICEF framework appears to be most used.

The WHO and UNICEF frameworks and other definitions found in the present scoping review share some similarities. They highlight that life skills are necessary to interact with others and can be transferred between different contexts. In addition, a distinction is often made between categories of life skills, such as cognitive, personal, and interpersonal life skills. Taking into account these results as well as recent societal developments such as rapid technological advancements and generally worsened mental health since the Covid-19 pandemic, we propose the following definition for the concept of life skills for emerging adults: Life skills are cognitive, personal, and interpersonal skills necessary to promote good mental health, autonomous living, to regulate oneself, and to successfully interact with other members of society across different contexts (e.g., family or work) and settings (e.g., home or office).

### Lessons learned for life skills development and transfer

While in younger age groups (e.g., adolescents) life skills development and transfer has been heavily researched in sports contexts (Danish and Nellen, 1997; Petitpas et al., 2004; Theokas et al., 2008; Jacobs and Wright, 2021), the sports context is much less salient in life skills studies for emerging adults. Camiré’s (2022) model of life skills teaching -developed in the context of youth sports coaching- may therefore not be directly applicable, but it appears that most life skills studies take a rather implicit approach as only a minority of studies were intervention studies and even within the intervention studies, specific life skills were lacking regularly. It is possible that the underlying assumption in a majority of studies is that life skills are learned as a byproduct, but this remains speculative. Along the normative/transformative axis it can be deduced that genuinely transformative approaches to life skills development in emerging adults are absent with the exception of Mmusi and van Breda (2017), which was also the only study discussing specific cognitive, personal, and interpersonal transfer components. It may be further explored to which extent the transfer components these authors found (recall, contextual flexibility, personal challenges, upbringing, purpose/perceived usefulness, thoughtfulness, willingness, internalization, outcome expectation, perceived ability to use skill, and reflective living) are applicable in contexts other than South African foster care.

Furthermore, the results give little impetus on actionable insides regarding life skills development and transfer in non-vulnerable emerging adults groups. A notable exception is the study by Güçlü et al. (2022) who investigated emerging adults’ perceived life skills through a self-developed questionnaire in a group of Turkish university students. Their results revealed that the level of perceived life skills was significantly predicted by self-regulation (i.e., regulation of emotions, thoughts, and behaviors) and autonomy supportive behaviors by parents and teachers. These results may still be further explored in other settings for life skills development interventions, for example to assess whether self-regulation may be considered an overarching life skill that could facilitate the internalization of other skills.

A final lesson regarding life skills development that can be learned from the results of the scoping review is that life skills studies tend to be reactive rather than pro-active. The UNICEF framework stresses the empowering nature of life skills to live productive, meaningful and fulfilling lives. The current results, however, indicate that life skills studies mostly focus on reactively teaching life skills once a problem (e.g., developmental or contextual) has occurred. This is highlighted by the fact that the vast majority of studies dealt with vulnerable populations and life skills were proposed for these specific contexts, whereas very few studies discussed pro-active life skills teaching for non-vulnerable populations.

### Future research

Based on the results of the present scoping review, we propose the following agenda items for future research regarding life skills development and transfer in emerging adults. First, a clear conceptual framework has to be derived. As the results have shown, few studies clarify the theoretical background. The UNICEF framework was published over a decade ago in 2012 and society has experienced some fundamental changes since. First, major technological advancements have rapidly changed society, with near universal internet and social media accessibility at our fingertips. These developments have rapidly changed the ways in which we communicate and interact with one another. A plethora of research indicates that internet access and social media in particular have a fundamental influence on the self-image, self-esteem and general mental health, with adolescents and emerging adults experiencing the greatest impact (Richards et al., 2015; Hawi and Samaha, 2017; Rounsfell et al., 2020; Bettmann et al., 2021). Second, as outlined previously, the Coronavirus pandemic has had major mental health implications for different age and ethnic groups (Shuja et al., 2020; Roy et al., 2021; Saltzman et al., 2021; De France et al., 2022). In light of these changes, it may be necessary to develop an updated framework. In addition, it would be beneficial to derive a life skills framework for the particular age group of emerging adults. An interesting starting point for such a framework, as well as a way of categorizing future studies, may be the framework proposed by Camiré (2022). The adaptation of that framework for studies outside of the youth sport context is therefore a recommended avenue for future research.

The second agenda item concerns research regarding the cognitive, personal, and interpersonal components of life skills transfer is. As mentioned previously, of the few studies mentioning life skills transfer, only one discussed such transfer components. In other words, almost no actionable insights regarding life skills transfer in emerging adults can be derived from the current state of the research. This result was somewhat unexpected because life skills transfer has received considerable attention in sport settings, particularly in vulnerable populations (see, e.g., the systematic review by ter Harmsel-Nieuwenhuis et al., 2022) and youth populations (Pierce et al., 2019; Martin and Camiré, 2020; Jacobs and Wright, 2021). It is, however, unclear whether or how these insights are applicable to typically developing emerging adults. Thus, future research should put more emphasis on the specific life skills transfer components in non-vulnerable emerging adults, through quantitative questionnaire studies as well as qualitative interviews and focus groups.

A third avenue for future research concerns the development of a curriculum for life skills development in non-vulnerable emerging adults based on the first two agenda items. Such a curriculum could for example be implemented in coursework at universities to better prepare students for the transition to adulthood: “life skills training should include elements to enhance the development of individual coping strategies to apply when life feels tough and when the body is experiencing stress reactions” (Hellström and Beckman, 2021, p. 1). A possible curriculum should recognize the volatility of emerging adulthood to train students in life skills that are applicable across a diverse range of contexts and explicitly address the transfer process. In that context, it could also be researched which social expectations are linked to developmental tasks such as acquiring life skills, and if life skills can be used as a preventive measure for developing mental health issues.

A final avenue for future research could address the question: what efforts need to be made to increase the readiness of emerging adults to achieve the life skills needed at that age? This question is separate from the question which specific life skills interventions are used to train life skills. It is believed that the learning outcomes for life skills will be more successful if emerging adults are intrinsically motivated. From an evolutionary perspective “intrinsic motivations drive the acquisition of knowledge and skills that contribute to produce behaviors that increase fitness” (Baldassarre, 2011, p. 1) to one’s environment. Intrinsically motivated emerging adults would therefore possibly better adapt to their changing environments. Addressing motivation would also put stronger emphasis on a proactive approach to life skills outside of the mental health context. As outlined by The World Bank (2013), life skills are not only important for (mental) health outcomes, but also for education and labor market outcomes. These outcomes are considered paramount for emerging adults to manage the transition to a successful adult life. Addressing motivation and outcomes beyond the mental health context is thus considered an important avenue for future research.

In addition to these avenues for future research, future life skills studies for emerging adults should improve on reporting. In the vast majority of studies, it was unclear which theoretical framework was used and how exactly the term life skills was defined. A crucial recommendation for future studies is therefore to report these characteristics to improve the methodological quality, transparency, and reproducibility.

## Limitations

We systematically searched four databases (Scopus, PsycInfo, Pubmed, and Web of Science). Therefore, the final sample included in the present scoping review is necessarily limited to journal articles which are indexed in those four databases. In addition, we specified in the search query that articles must include either of the following terms: emerging adult or adulthood, young adult, or young people. Articles that may have fallen within the desired age range, but did not explicitly mention one of these terms, may therefore not have come up during the search. However, specifying these terms increased the probability of finding articles that focused explicitly on the transition period. Alternatively, it would have been possible to set an age filter for the search, but that would have excluded articles that do not specify the age rage in the abstract or keywords and such age filters may work differently between the various databases.

## Conclusion

Life skills have received much attention in the academic literature, but the plethora of the research focuses on children and adolescents. Emerging adulthood is a sensitive and volatile life period during that can be the onset of many chronic psychological disorders. The COVID-19 pandemic has further detrimentally impacted mental health in emerging adults. Life skills have been proposed as a catalyst for successfully mastering the transition to adulthood. However, research on life skills development in emerging adults is mostly limited to vulnerable populations. In addition, transfer of life skills between contexts has received virtually no attention at all. It is concluded that life skills development and transfer in typically developing emerging adults are understudied and future research needs to develop a framework to address this gap.

## Data availability statement

Publicly available datasets were analyzed in this study. This data can be found here: Open Science Framework: https://osf.io/gmk8w/.

## Author contributions

RT: Conceptualization, Data curation, Formal analysis, Investigation, Methodology, Visualization, Writing – original draft, Writing – review & editing. PG: Conceptualization, Methodology, Validation, Writing – review & editing. WJ: Conceptualization, Methodology, Project administration, Supervision, Writing – review & editing. EV: Conceptualization, Funding acquisition, Project administration, Supervision, Validation, Writing – review & editing.

## Footnotes

^1^First published online in 2021.

## Supplementary material

Supplementary Appendix A Number of hits for the search term “life skill*” in PubMed per year 2010–2022.
