# Supplementary figures and images for: A scoping review of life skills development and transfer in emerging adults
Source: Front Psychol. 2023 Nov 16;14:1275094. doi: 10.3389/fpsyg.2023.1275094 (PMC10690614; doi:10.3389/fpsyg.2023.1275094)

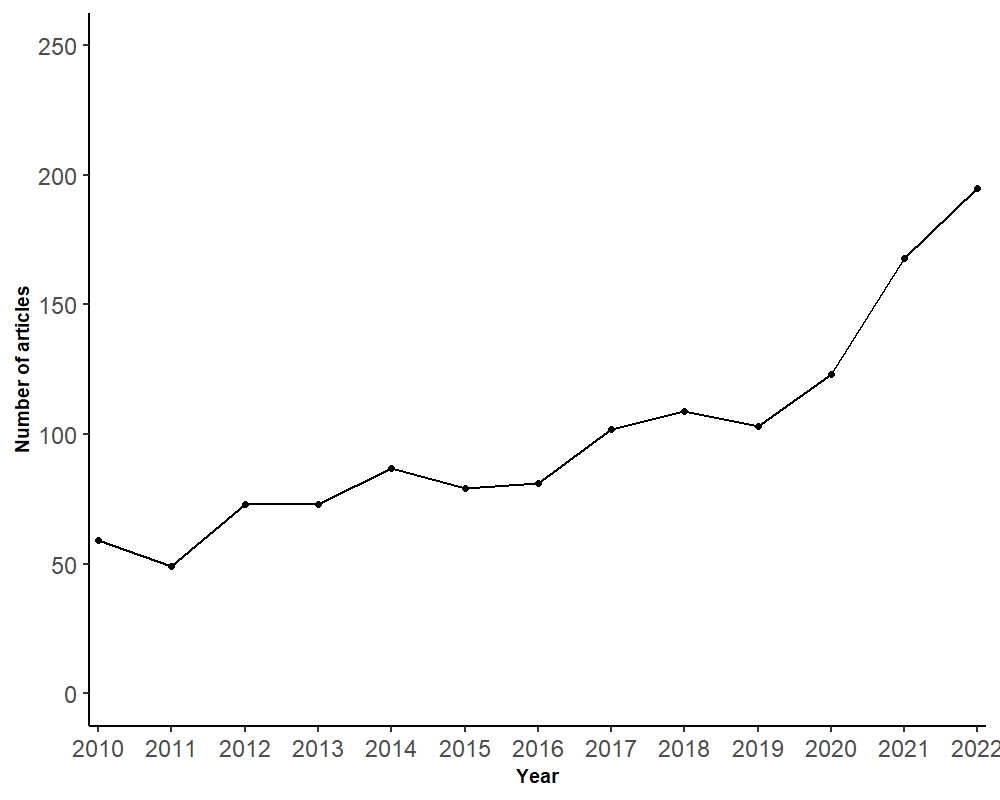

Supplement: Supplementary Appendix A — Number of hits for the search term “life skill*” in PubMed per year 2010–2022. [file Image_1.jpeg]
