# Supplementary material for: A scoping review of life skills development and transfer in emerging adults
Source: Front Psychol. 2023 Nov 16;14:1275094. doi: 10.3389/fpsyg.2023.1275094 (PMC10690614; doi:10.3389/fpsyg.2023.1275094)
Supplement: Supplementary file 2 [file Data_Sheet_1.docx]

**Appendix B: Search query per data base**

**Web of Science**

((((((TI=(((“life skill*” OR “lifeskill*") AND ("young adult*" OR "emerging adult*" OR “young people”) )))) OR AB=(((“life skill*” OR “lifeskill*") AND ("young adult*" OR "emerging adult*" OR “young people”) )))) OR AK=(((“life skill*” OR “lifeskill*") AND ("young adult*" OR "emerging adult*" OR “young people”) )))) AND LA=(English)

Applied filter: Date 2010-01-01 – 2022-10-31

**Scopus**

TITLE-ABS-KEY("life skill*" OR "lifeskill*") AND TITLE-ABS-KEY("young adult*" OR "emerging adult*" OR "young people") AND ( LIMIT-TO ( PUBYEAR,2022) OR LIMIT-TO ( PUBYEAR,2021) OR LIMIT-TO ( PUBYEAR,2020) OR LIMIT-TO ( PUBYEAR,2019) OR LIMIT-TO ( PUBYEAR,2018) OR LIMIT-TO ( PUBYEAR,2017) OR LIMIT-TO ( PUBYEAR,2016) OR LIMIT-TO ( PUBYEAR,2015) OR LIMIT-TO ( PUBYEAR,2014) OR LIMIT-TO ( PUBYEAR,2013) OR LIMIT-TO ( PUBYEAR,2012) OR LIMIT-TO ( PUBYEAR,2011) OR LIMIT-TO ( PUBYEAR,2010) ) AND ( LIMIT-TO ( LANGUAGE,"English" ) )

**PubMed**

((("life skill*"[Title/Abstract] OR "lifeskill*"[Title/Abstract]) AND ("young adult*"[Title/Abstract] OR "emerging adult*"[Title/Abstract] OR "young people"[Title/Abstract] "young people")) AND (English[Language])) AND (("2010/01/01"[Date - Create] : "2022/10/31"[Date - Create]))

**PsycInfo**

tiab("young adult" OR "young adulthood" OR "young adults" OR "emerging adult" OR "emerging adulthood" OR "emerging adults" OR "young people") AND tiab("life skill*" OR "lifeskill*")

Applied filters: English language, 18 Yrs & older, Publication date: 01/01/2022 – 10/31/2022
